# Human Inborn Errors of Immunity: 2019 Update of the IUIS Phenotypical Classification

**DOI:** 10.1007/s10875-020-00758-x

**Published:** 2020-02-11

**Authors:** Aziz Bousfiha, Leila Jeddane, Capucine Picard, Waleed Al-Herz, Fatima Ailal, Talal Chatila, Charlotte Cunningham-Rundles, Amos Etzioni, Jose Luis Franco, Steven M Holland, Christoph Klein, Tomohiro Morio, Hans D. Ochs, Eric Oksenhendler, Jennifer Puck, Troy R. Torgerson, Jean-Laurent Casanova, Kathleen E. Sullivan, Stuart G. Tangye

**Affiliations:** 1 Laboratoire d’Immunologie Clinique, d’Inflammation et d’Allergy LICIA, Faculty of Medecine and Pharmacy, King Hassan II University, Casablanca, Morocco; 2 Clinical Immunology Unit, Pediatric Infectiouse Disease Departmentn Children’s Hospital, Ibn Rochd University Hospital, Casablanca, Morocco; 3 Laboratoire national de référence, University Mohamed VI of Health Sciences, Casablanca, Morocco; 4 Study Center for Primary Immunodeficiencies, Necker Hospital for Sick Children, APHP, Paris University, Paris, France; 5 Laboratory of Lymphocyte Activation and Susceptibility to EBV, INSERM UMR1163, Imagine Institute, Necker Hospital for Sick Children, Paris University, Paris, France; 6 Department of Pediatrics, Faculty of Medicine, Kuwait University, Kuwait City, Kuwait; 7 Division of Immunology, Children’s Hospital Boston, Boston, USA; 8 Departments of Medicine and Pediatrics, Mount Sinai School of Medicine, New York, USA; 9 Ruth’s Children’s Hospital-Technion, Haifa, Israel; 10 Grupo de Inmunodeficiencias Primarias, Facultad de Medicina, Universidad de Antioquia UdeA, Medellin, Colombia; 11 Laboratory of Clinical Immunology & Microbiology, National Institute of Allergy and Infectious Diseases, National Institutes of Health, Bethesda, USA; 12 Dr von Hauner Children’s Hospital, Ludwig-Maximilians-University Munich, Munich, Germany; 13 Department of Pediatrics and Developmental Biology, Tokyo Medical and Dental University (TMDU), Tokyo, Japan; 14 Department of Pediatrics, University of Washington and Seattle Children’s Research Institute, Seattle, USA; 15 Department of Clinical Immunology, Hôpital Saint-Louis, APHP, University Paris Diderot, Sorbonne Paris, Cité, Paris, France; 16 Department of Pediatrics, University of California San Francisco and UCSF Benioff Children’s Hospital, San Francisco, USA; 17 St. Giles Laboratory of Human Genetics of Infectious Diseases, Rockefeller Branch, The Rockefeller University, New York, USA; 18 Howard Hughes Medical Institute, New York, USA; 19 Laboratory of Human Genetics of Infectious Diseases, Necker Branch, INSERM UMR1163, Imagine Institute, Necker Hospital for Sick Children, Paris University, Paris, France; 20 Pediatric Hematology-Immunology Unit, Necker Hospital for Sick Children Assistance Publique-Hôpitaux de Paris (APHP), Paris, France; 21 Division of Allergy Immunology, Department of Pediatrics, The Children’s Hospital of Philadelphia, University of Pennsylvania Perelman School of Medicine, Philadelphia, USA; 22 Garvan Institute of Medical Research, Darlinghurst, Australia; 23 St Vincent’s Clinical School, Faculty of Medicine, UNSW, Sydney, Australia

**Keywords:** IUIS, primary immune deficiency, inborn errors of immunity, immune dysregulation, autoinflammatory disorders, classification

## Abstract

Since 2013, the International Union of Immunological Societies (IUIS) expert committee (EC) on Inborn Errors of Immunity (IEI) has published an updated phenotypic classification of IEI, which accompanies and complements their genotypic classification into ten tables. This phenotypic classification is user-friendly and serves as a resource for clinicians at the bedside. There are now 430 single-gene IEI underlying phenotypes as diverse as infection, malignancy, allergy, autoimmunity, and autoinflammation. We herein report the 2019 phenotypic classification, including the 65 new conditions. The diagnostic algorithms are based on clinical and laboratory phenotypes for each of the ten broad categories of IEI.

## Introduction

Human inborn errors of immunity (IEI) are caused by monogenic germline mutations resulting in loss or gain of function of the encoded protein. They can be dominant or recessive, autosomal or X-linked, and with complete or incomplete penetrance. They manifest as increased susceptibility to a broad or narrow spectrum of infectious diseases, as well as a growing diversity of autoimmune, autoinflammatory, allergic, and/or malignant phenotypes. They now comprise 406 distinct disorders with 430 different gene defects listed in the 2019 International Union of Immunological Societies (IUIS) classical classification [1]. If most IEI are individually rare, they are collectively more common than generally thought [2].

The (IUIS) expert committee on IEI proposes every other year a genotypic classification of all these disorders [1], which facilitates both research on, and diagnosis of, these conditions worldwide. This classification is organized in ten tables, each of which groups IEI sharing a given pathogenesis. However, with the growing number of IEI included in this catalog, these tables are not always easy to use at the bedside. We thus reported from 2013 onward a more user-friendly classification adapted for the clinician, based on the clinical and laboratory features observed in these patients. This phenotypic classification proved to be as popular as the genotypic classification (15 k vs 12 k downloads on publisher site) [3] and has been adapted in a smartphone application [4].

Here, we present an update of the phenotypic classification of IEI, based on the 2019 IEI classical classification [1]. This tree-based decision-making process is aimed to physicians, regardless of their familiarity with IEI. It aims at helping them to reach a diagnosis based on simple clinical and biological phenotypes.

## Methodology

We included in our figures all disorders indexed in the 2019 update of the IUIS IEI classification [1]. A phenotypic algorithm was assigned to each of the ten main groups of the classification and the same color was used for each group of similar conditions. Given the high number of diseases, several categories have been split since last update [3] in two sub-figures to be more informative.

Disease names are presented in red and genes in bold italic. An asterisk is added to highlight extremely rare disorders (less than 10 reported cases to date). However, the reader should keep in mind that some genes have been very recently described and that true prevalence is unknown. A double asterisk is added when only one case or one kindred has been reported to date. In these cases, it is difficult to confirm than observed phenotype would be reproducible in other patients carrying the same defect, or if it is an exception.

## Results

Algorithms for the 2019 update of IUIS phenotypical classification are presented in Figs. 1, 2, 3, 4, 5, 6, 7, 8, 9, and 10.

**Fig. 1:**
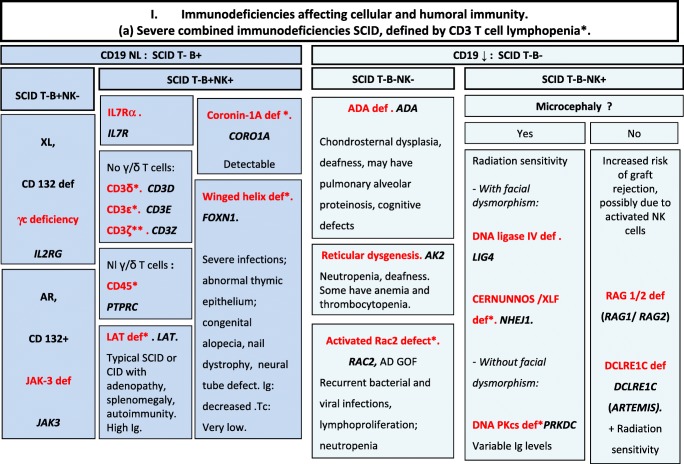

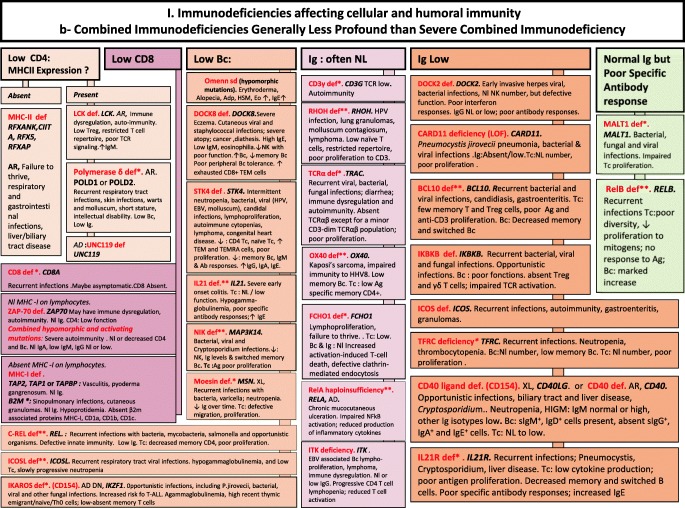
Immunodeficiencies affecting cellular and humoral immunity. **a** Severe combined immunodeficiencies defined by T cell lymphopenia. **b** Combined immunodeficiencies. * T cell lymphopenia in SCID is defined by CD3+ T cells < 300/μL. AD autosomal dominant transmission, ADA adenosine deaminase, Adp adenopathies, Ag antigen, AR autosomal recessive transmission, β2m bêta-2 microglobulin, Bc B cells, CBC complete blood count, CD cluster of differentiation, CVID common variable immunodeficiency, def deficiency, EBV Epstein-Barr virus, Eo eosinophilia, GOF gain-of-function mutation, HHV8 human herpes virus 8, HIGM hyper IgM syndrome, HPV human papillomavirus, HSM hepatosplenomegaly, Ig immunoglobulins, MHC major histocompatibility complex, Nl normal, NK natural killer, SCID severe combined immunodeficiency, Tc T cells, TCR T cell receptor, Treg regulatory T cells, XL X-linked transmission

**Fig. 2:**
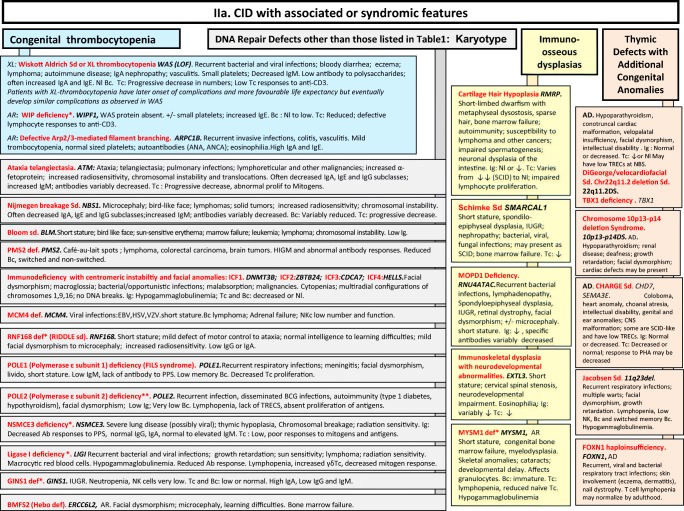

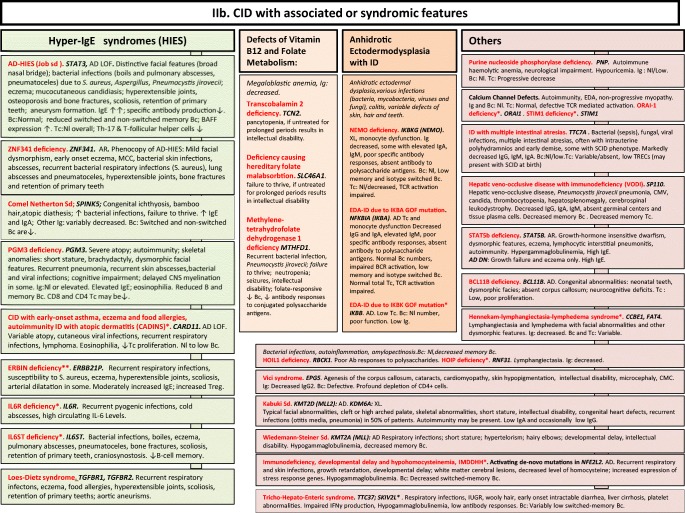
**a**, **b** CID with associated or syndromic features. Ab antibody, AD autosomal dominant transmission, AD DN autosomal dominant transmission with dominant negative effect, ANA anti-nuclear antibodies, ANCA anti-neutrophil cytoplasm antibodies, AR autosomal recessive transmission, Bc B cells, BCG bacillus Calmette-Guerin, BCR B cell receptor, CD cluster of differentiation, CID combined immunodeficiency of T and B cells, CMV cytomegalovirus, CNS central nervous system, def deficiency, DNA deoxyribonucleic acid, EBV Epstein-Barr virus, EDA anhidrotic ectodermal dysplasia, GOF gain-of-function, HIES hyper IgE syndrome, FILS facial dysmorphism, immunodeficiency, livedo and short stature, ID immunodeficiency, Ig immunoglobulins, IL-6 interleukin-6, IUGR intrauterine growth retardation, LOF loss-of-function, MCC mucocutaneous candidiasis, Nl normal, NK natural killer, PHA phytohemagglutinin, PPS polysaccharides, SCID severe combined immunodeficiency, sd syndrome, Tc T cells, TCR T cell receptor, TREC T cell receptor excision circle, XL X-linked transmission

**Fig. 3:**
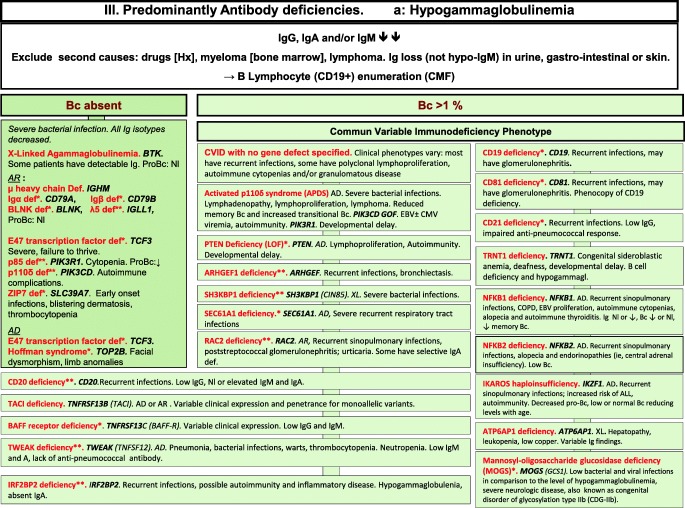

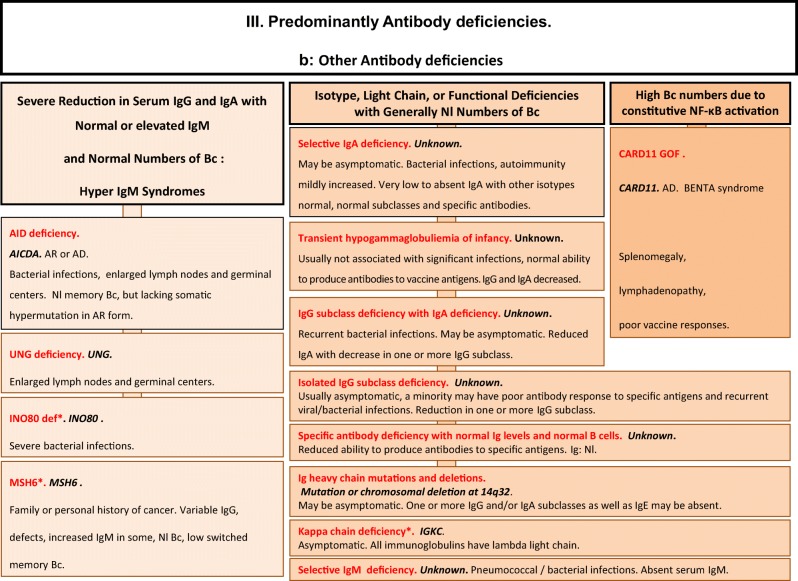
Predominantly antibody deficiencies. **a** Hypogammaglobulinemias. **b** Other antibody deficiencies. AD autosomal dominant transmission, AR autosomal recessive transmission, Bc B cells, BENTA B cell expansion with NF-κB and T cell anergy, CD cluster of differentiation, CMF flow cytometry, COPD chronic obstructive pulmonary disease, def deficiency, EBV Epstein-Barr virus, GOF gain-of-function, Hx patient history, Ig immunoglobulins, Nl normal, XL X-linked transmission

**Fig. 4:**
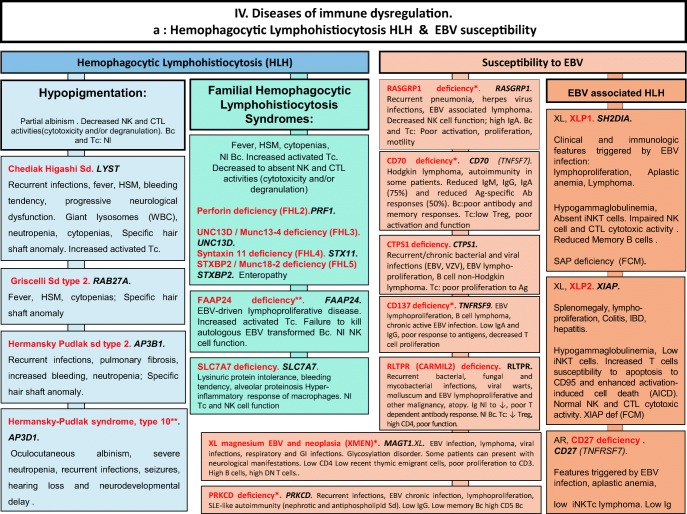

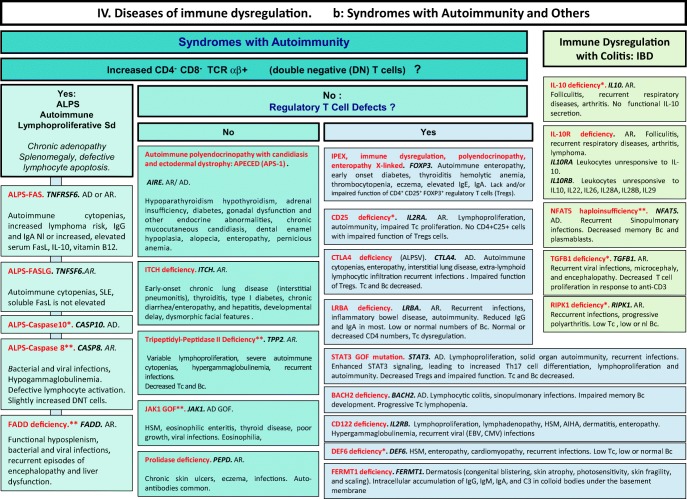
Diseases of immune dysregulation. **a** Hemophagocytic lymphohistiocytosis. **b** Other diseases of immune dysregulation. Ab antibody, AD autosomal dominant transmission, Ag antigen, AIHA autoimmune hemolytic anemia, ALPS autoimmune lymphoproliferative syndrome, APS autoimmune polyendocrinopathy syndrome, AR autosomal recessive transmission, Bc B cells, CD cluster of differentiation, CMF flow cytometry, CTL cytotoxicT lymphocytes, def deficiency, DNT double negative T cells, EBV Epstein-Barr virus, FHL familial hemophagocytic lymphohistiocytosis, GOF gain-of-function, HLH hemophagocytic lymphohistiocytosis, (H)SM (hepato)splenomegalia, IBD inflammatory bowel disease, Ig immunoglobulin, IL-10 interleukin-10, LOF loss-of-function, iNKT invariant NKT cells, NK natural killer cells, Nl normal, sd syndrome, SLE systemic lupus erythematous disease, Tc T cells, TCR T cell receptor, XL X-linked transmission

**Fig. 5:**
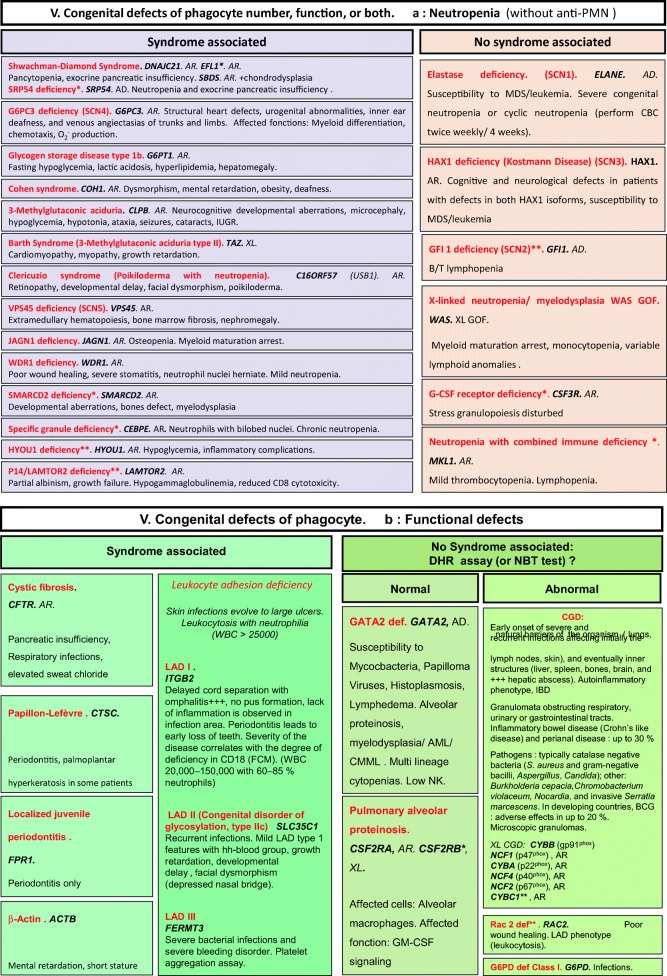
Congenital defects of phagocyte number, function, or both. **a** Neutropenia. **b** Functional defects of phagocytes. AD autosomal dominant transmission, AML acute myeloid leukemia, AR autosomal recessive transmission, BCG bacillus Calmette-Guerin, CD cluster of differentiation, CGD chronic granulomatous disease, CMF flow cytometry, CMML chronic myelomonocytic leukemia, def deficiency, DHR dihydrorhodamine-1,2,3, GM-CSF granulocytes/monocytes colony stimulation factor, GOF gain-of-function, IBD inflammatory bowel disease, IUGR intrauterine growth retardation, LAD leukocyte adhesion deficiency, MDS myelodysplasia, NBT nitroblue of tetrazolium, NK natural killer cells, WBC white blood cells, XL: X-linked transmission

**Fig. 6:**
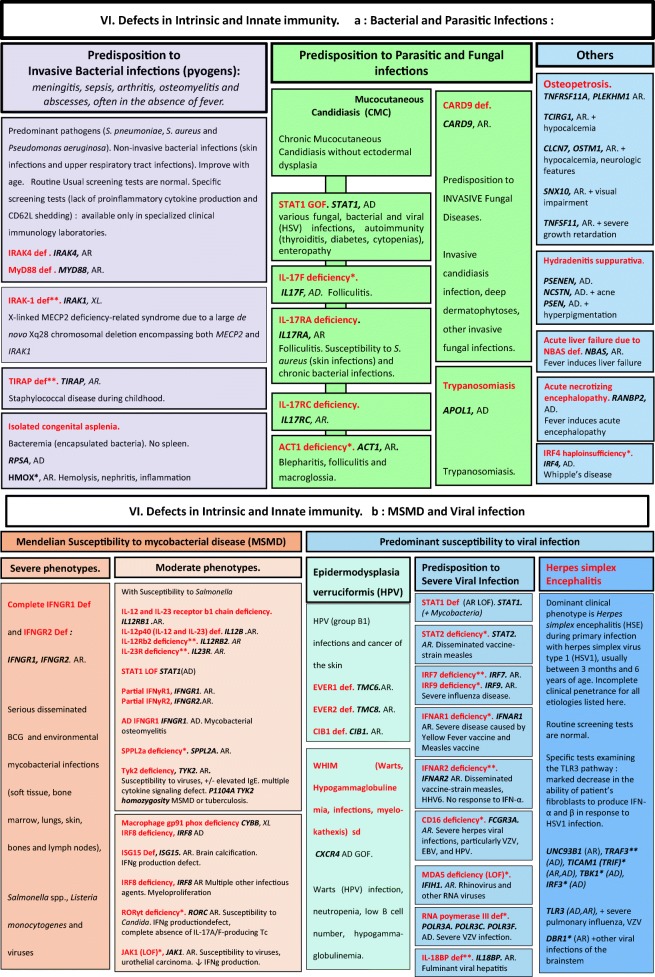
Defects in intrinsic and innate immunity. **a** Bacterial and parasitic infections. **b** MSMD and viral infection. AD autosomal dominant transmission, AR autosomal recessive transmission, BCG bacillus Calmette-Guerin, CD cluster of differentiation, CMC chronic mucocutaneous candidiasis, EBV Epstein-Barr virus, GOF gain-of-function, IFNg interferon gamma, HHV6 human herpes virus type 6, HPV human papillomavirus, HSV herpes simplex virus, LOF loss-of-function, MSMD Mendelian susceptibility to mycobacterial disease, NK natural killer cells, RNA ribonucleic acid, sd syndrome, Tc T cells, TLR3 Toll-like receptor type 3, VZV varicella zoster virus, XL X-linked transmission

**Fig. 7:**
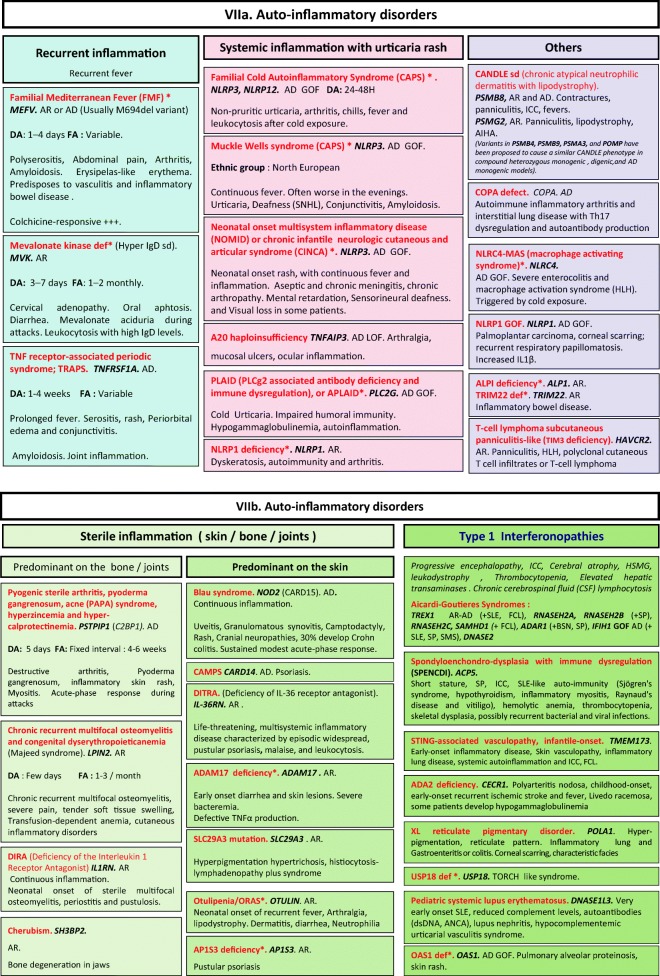
**a**, **b** Autoinflammatory disorders. AD autosomal dominant transmission, ANCA anti-neutrophilic cytoplasmic autoantibody, AR autosomal recessive transmission, BSN bilateral striatal necrosis, CAPS cryopyrin-associated periodic syndrome, DA duration of acute inflammation episode, dsDNA double-stranded deoxyribonucleic acid, FA frequency of acute inflammation episode, FCL familial chilblain lupus, GOF gain-of-function, HLH hemophagocytic lymphohistiocytosis, HSM hepatosplenomegalia, ICC intracranial calcifications, IL interleukin, LOF loss-of-function, sd syndrome, SLE systemic lupus erythematosus, SMS Singleton-Merten syndrome, SNHL sensorineural hearing loss, SP spastic paraparesis, TORCH toxoplasmosis, other, rubella, cytomegalovirus, and herpes infections

**Fig. 8:**
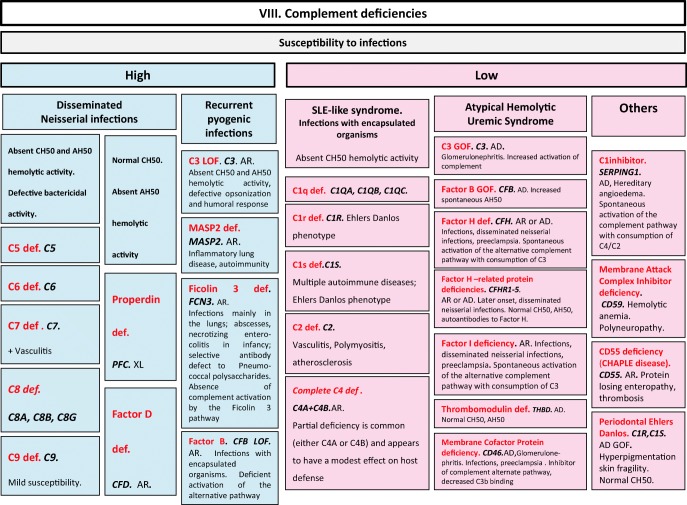
Complement deficiencies. AD autosomal dominant transmission, AH50 alternate pathway hemolytic activity, AR autosomal recessive transmission, CH50 complement hemolytic activity, def deficiency, GOF gain-of-function, LOF loss-of-function, sd syndrome, SLE systemic lupus erythematosus, XL X-linked transmission

**Fig. 9:**
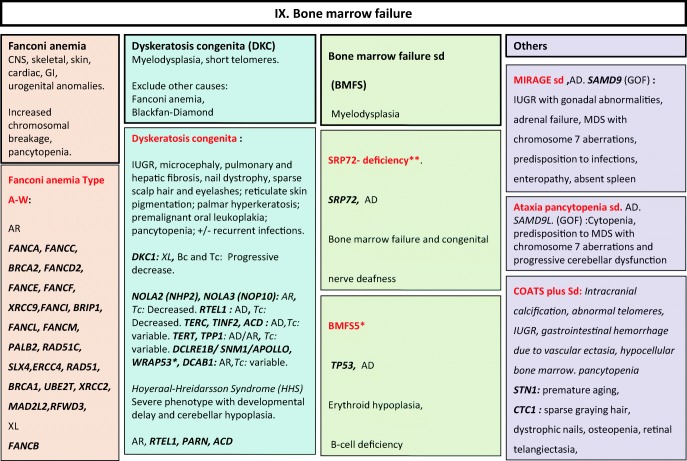
Bone marrow failure disorders. AD autosomal dominant transmission, AR autosomal recessive transmission, Bc B cells, BMFS bone marrow failure syndrome, CNS central nervous system, DKC dyskeratosis congenita, GI gastrointestinal, GOF gain-of-function, IUGR intrauterine growth retardation, MDS myelodysplasia, sd syndrome, Tc T cells, XL X-linked transmission

**Fig. 10:**
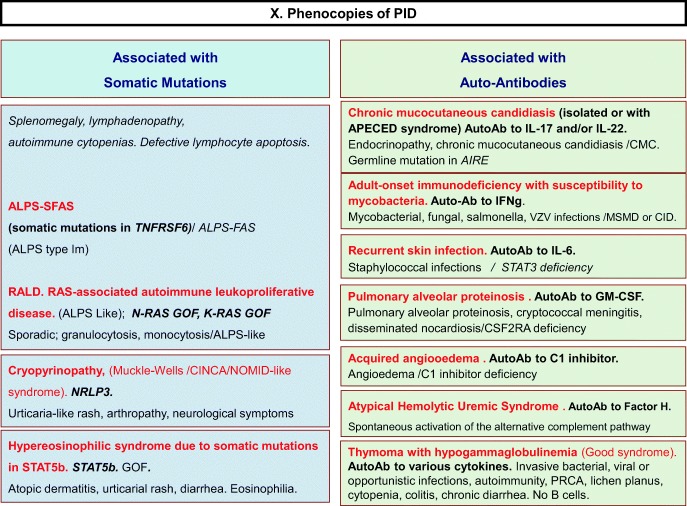
Phenocopies of PID. ALPS autoimmune lymphoproliferative syndrome, AutoAb autoantibodies, CID combined immunodeficiency, CMC chronic mucocutaneous candidiasis, GOF gain-of-function, MSMD Mendelian susceptibility to mycobacterial disease, PRCA pure red cell aplasia

## Discussion

These algorithms are aimed to guide clinicians to diagnose patients presenting typical phenotype. However, readers should be aware of the limitations of such a work.

More and more reports show a spectrum of atypical presentations related to hypomorphic mutations of those genes. Omenn syndrome (OMIM #603554) is a good example of such an atypical presentation, as well as “leaky SCID” and RAG deficiency spectrum [5].

Moreover, readers should be extremely cautious with descriptions of disease when only one patient or kindred have been reported. We are aware that these reports may not reflect the typical phenotype of such defects, but the exception; however, we thought that it was needed to be mentioned in these classifications.

## Conclusion

This phenotypic classification of IEI forms a diagnostic resource, aimed to complement the 2019 IUIS genotypic classification. These figures serve as diagnostic orientation tools for patients with any of the typical phenotypic presentations of IEI, whether clinical or biological. They were designed for, and will hopefully be useful to physicians and biologists who are not experts in the field of IEI. We hope that these figures can help them reach a diagnosis of IEI when encountering patients whose clinical or biological phenotypes are evocative of IEI.
